# Cumulative Deleterious Effects of Tetrahydrocannabinoid (THC) and Ethanol on Mitochondrial Respiration and Reactive Oxygen Species Production Are Enhanced in Old Isolated Cardiac Mitochondria

**DOI:** 10.3390/ijms25031835

**Published:** 2024-02-02

**Authors:** Anne-Laure Charles, Anne Charloux, Thomas Vogel, Jean-Sébastien Raul, Michel Kindo, Valérie Wolff, Bernard Geny

**Affiliations:** 1 Biomedicine Research Center of Strasbourg (CRBS), UR 3072, “Mitochondria, Oxidative Stress and Muscle Plasticity”, University of Strasbourg, 67000 Strasbourg, France; anne.laure.charles@unistra.fr (A.-L.C.); anne.charloux@chru-strasbourg.fr (A.C.); thomas.vogel@chru-strasbourg.fr (T.V.); michel.kindo@chru-strasbourg.fr (M.K.); valerie.wolff@chru-strasbourg.fr (V.W.); 2 Faculty of Medicine, University of Strasbourg, 67000 Strasbourg, France; jean-sebastien.raul@chru-strasbourg.fr; 3 Department of Physiology and Functional Explorations, University Hospital of Strasbourg, 67091 Strasbourg, France; 4 Geriatrics Department, University Hospital of Strasbourg, 67091 Strasbourg, France; 5 Toxicology Laboratory, Institute of Legal Medicine, Faculty of Medicine, University of Strasbourg, 67000 Strasbourg, France; 6 Cardiovascular Surgery Department, University Hospital of Strasbourg, 67091 Strasbourg, France; 7 Neuro-Vascular Department, University Hospital of Strasbourg, 67098 Strasbourg, France

**Keywords:** tetrahydrocannabinoid, cannabis, marijuana, heart, mitochondria, oxidative stress, aging, alcohol, ethanol

## Abstract

Delta 9 tetrahydrocannabinol (THC), the main component of cannabis, has adverse effects on the cardiovascular system, but whether concomitant ethanol (EtOH) and aging modulate its toxicity is unknown. We investigated dose responses of THC and its vehicle, EtOH, on mitochondrial respiration and reactive oxygen production in both young and old rat cardiac mitochondria (12 and 90 weeks). THC dose-dependently impaired mitochondrial respiration in both groups, and such impairment was enhanced in aged rats (−97.5 ± 1.4% vs. −75.6 ± 4.0% at 2 × 10^−5^ M, and IC50: 0.7 ± 0.05 vs. 1.3 ± 0.1 × 10^−5^ M, *p* < 0.01, for old and young rats, respectively). The EtOH-induced decrease in mitochondrial respiration was greater in old rats (−50.1 ± 2.4% vs. −19.8 ± 4.4% at 0.9 × 10^−5^ M, *p* < 0.0001). Further, mitochondrial hydrogen peroxide (H_2_O_2_) production was enhanced in old rats after THC injection (+46.6 ± 5.3 vs. + 17.9 ± 7.8%, *p* < 0.01, at 2 × 10^−5^ M). In conclusion, the deleterious cardiac effects of THC were enhanced with concomitant EtOH, particularly in old cardiac mitochondria, showing greater mitochondrial respiration impairment and ROS production. These data improve our knowledge of the mechanisms potentially involved in cannabis toxicity, and likely support additional caution when THC is used by elderly people who consume alcohol.

## 1. Introduction

Cannabis, the most frequently used psychoactive drug after alcohol and tobacco, is used annually by more than 180 million people worldwide, either for recreative or therapeutic purposes. It is now legalized in many states since cannabidiol, its primary nonpsychoactive compound, shows beneficial properties such as analgesia, anti-inflammation, etc. [1]. However, besides its well-known deleterious neurological and psychiatric effects, cannabis also induces adverse cardiovascular effects, including myocardial infarction, blood pressure changes, stroke, and peripheral vascular disease [2,3,4,5,6,7,8,9,10,11,12,13,14]. Particularly, when related to THC, myocardial infarction is characterized by a worse prognosis, and THC can now be considered as a risk factor for myocardial infarction [5,13] and stroke, often occurring in young people, with 18% of them presenting with significant long-term disability [9,11]. Recently, autopsy data supported that cannabis could trigger sudden death in predisposed subjects [14]. On the other hand, peripheral arterial disease, albeit linked to segmental narrowing of distal arteries with few collateral vessels, demonstrated potential reversibility [2,6,12]. 

The mechanisms underlying cannabis-related cardiovascular disease remain, at least, partly unexplained, but activation of the sympathetic system as well as inhibition of the parasympathetic autonomous nervous system, together with pro-coagulation activity and increased oxidative stress, are importantly involved [2,10,13]. Besides the earlier suggestions of arterial constriction and downregulation of myocardial function-related genes [13,15,16,17], cardiac mitochondrial dysfunction might be a co-factor participating in cannabis-induced cardiovascular disease. Indeed, as previously proposed in the setting of stroke [18], THC may increase a patient’s vulnerability to myocardial infarction. In this view, studies demonstrated a rapid absorption phase after marijuana smoking, and THC was measured in several tissues, including the heart [19,20,21,22]. Accordingly, THC impaired mitochondrial dysfunction in the brain [23], and similar data were observed when analyzing the effects of THC on several tissues, including the heart [24]. Thus, mitochondrial dysfunction might be a key factor in the cardiovascular effects of cannabis, as proposed by stroke data [18,23,24].

Interestingly, cardiovascular pathologies are more prevalent in older patients, but few studies have evaluated the impact of cannabis on the elderly despite its increasing use. In view of the growing population of marijuana users since the late 1960s, encounters with marijuana-related cardiovascular adversities may be silently on the rise [25,26]. Further, besides the high prevalence of cardiovascular risk factors in older people, aging per se and age-related enhancement of drug toxicity might increase mitochondrial susceptibility [2,27,28]. Further, although the interactive effects of THC and ethanol have been studied, showing reinforcement of consequences and synergistic effects on the perceived decreased ability to drive and on heart rate [29,30], such an association has not been precisely analyzed concerning cardiac mitochondrial function.

In the present study, we therefore determined the dose–response effects of delta 9 tetrahydrocannabinol (THC) associated with ethanol, the main cannabis psychoactive constituent, and of ethanol alone on cardiac mitochondria from both young and old rats, challenging the hypothesis that deleterious cardiac effects would be enhanced by concomitant ethanol and/or older age.

## 2. Results

### 2.1. Effect of Age on Baseline Mitochondrial Respiration and H_2_O_2_ Production 

**Figure 1:**
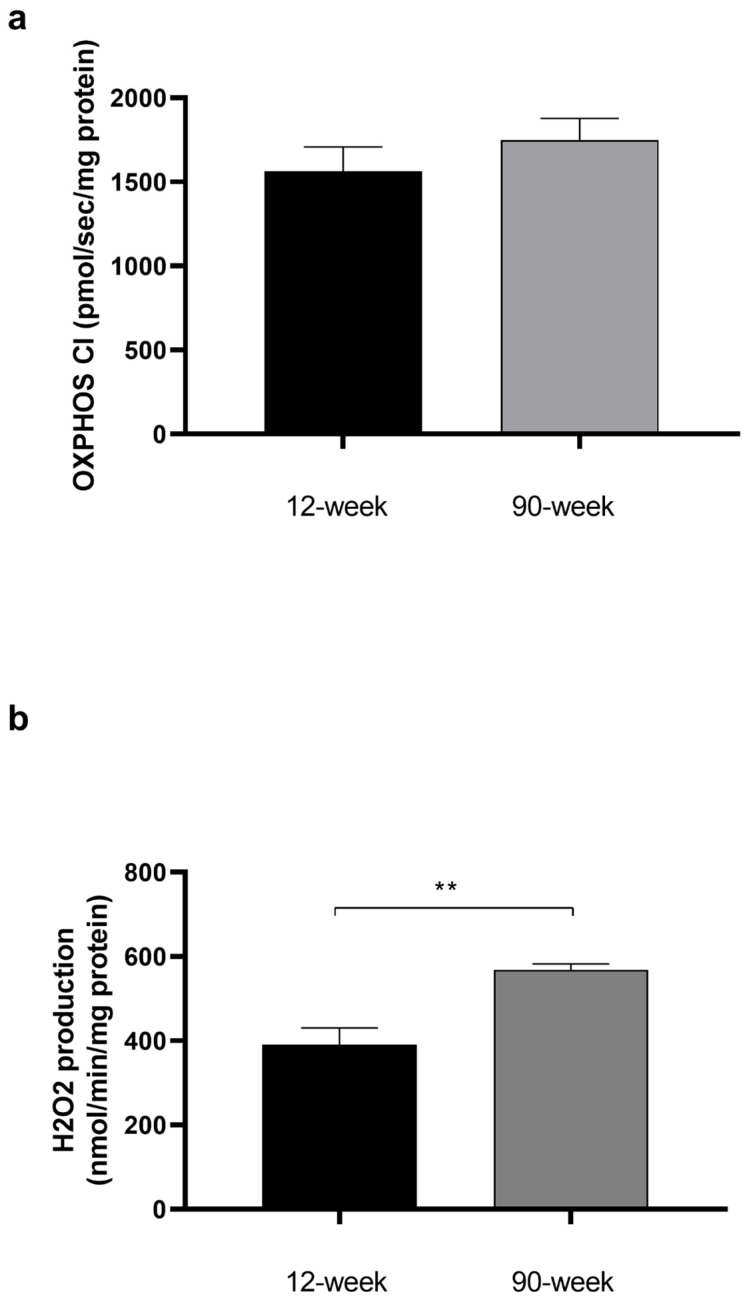
Effect of age on cardiac mitochondrial respiration and H_2_O_2_ production before THC or EtOH addition. (**a**): Mitochondrial respiration before THC or EtOH addition, corresponding to the 100% values shown in Figure 2a,b. n = 6 for 12-week group with 12 runs and n = 3 for 90-week group with 8 runs. (**b**): Mitochondrial H_2_O_2_ production. n = 2 for 12-week group with 4 runs and n = 3 for 90-week group with 8 runs. Values are means ± SEM. ** *p* < 0.01. OXPHOS CI: oxidative phosphorylation by the complex I.

Before THC or EtOH addition, mitochondrial respiration was similar in the two groups at 12 weeks and the two groups at 90 weeks. We therefore pooled the data at both 12 weeks and 90 weeks. The 100% values were 1566 ± 105 and 1766 ± 93 pmol/sec/mg, respectively (Figure 1a).

H_2_O_2_ values were also similar, so we pooled them by age. H_2_O_2_ production was significantly increased in aged cardiac mitochondria (391 ± 39 vs. 568 ± 14 nmol/min/mg, *p* < 0.01) (Figure 1b).

### 2.2. THC- and EtOH-Induced Impaired Mitochondrial Respiration Was Greater in Old as Compared to Young Rats

**Figure 2:**
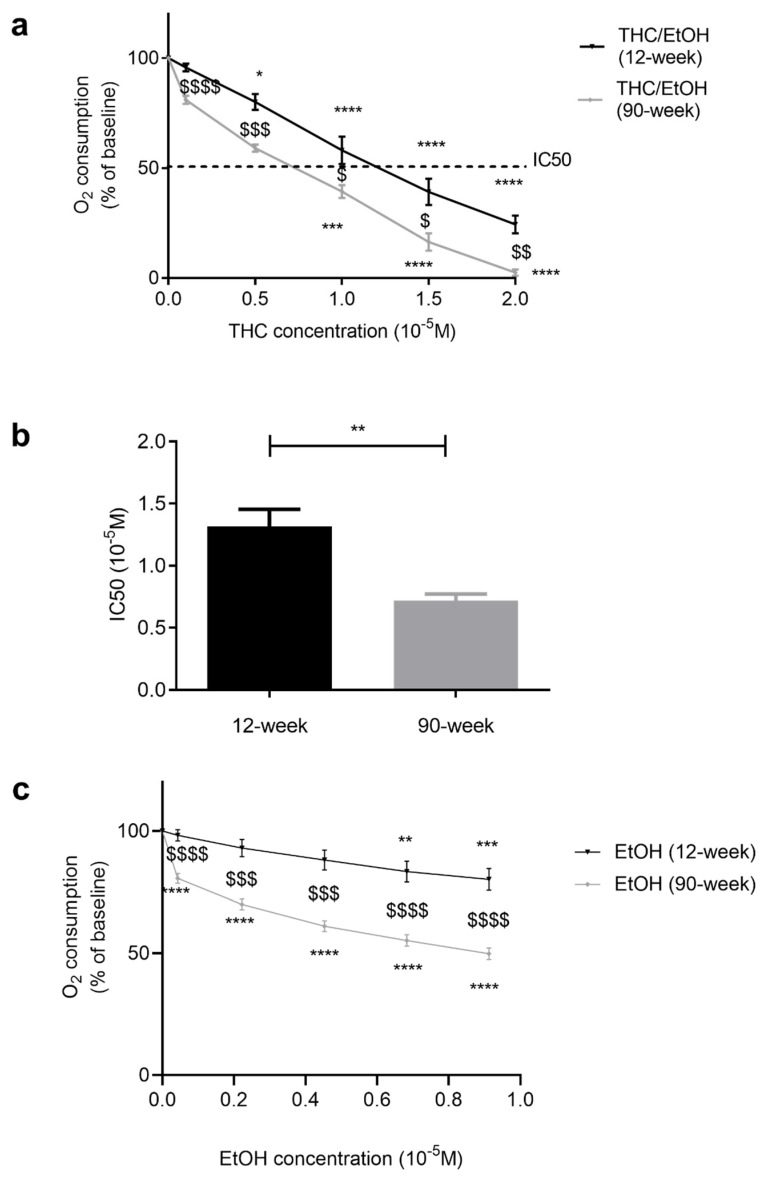
Dose–response effect of THC and EtOH on cardiac mitochondrial function, depending on age. (**a**): Cardiac mitochondrial respiration. Mitochondria were exposed to increasing THC doses. (**b**): IC50, THC needed to inhibit 50% of the maximal mitochondrial respiration. (**c**): Mitochondria were exposed to increasing EtOH doses corresponding to the increasing THC doses. Values are means ± SEM. n = 6 for 12-week group with 12 runs and n = 3 for 90-week group with 8 runs, * *p* < 0.05, ** *p* < 0.01, *** *p* < 0.001, **** *p* < 0.0001 vs. baseline. Comparisons between groups are denoted by $ *p* < 0.05, $$ *p* < 0.01, $$$ *p* < 0.001, $$$$ *p* < 0.0001. EtOH: ethanol. THC: tetrahydrocannabinoid.

In young adult rats, THC dose-dependently inhibited the cardiac mitochondrial respiration by −75.6 ± 4.0% at 2 × 10^−5^ M (*p* < 0.0001) (Figure 2a). In aged rats, the inhibition was even greater (−97.5 ± 1.4% at 2 × 10^−5^ M, *p* < 0.0001). Globally, increased age was associated with an enhanced mitochondrial inhibition.

Accordingly, IC50 (THC needed to inhibit 50% of the mitochondrial respiration) was obtained using less THC in old (0.7 ± 0.1 × 10^−5^ M) than in young rats (1.3 ± 0.1 × 10^−5^ M, *p* < 0.01) (Figure 2b).

EtOH alone, at the same dose used when acting as the solvent for THC, dose-dependently decreased mitochondrial respiration. Although relatively moderate, this decrease was significant at the higher doses in young rats (−19.8 ± 4.4% at 0.9 × 10^−5^ M, corresponding to the dose 2 × 10^−5^ M THC, *p* < 0.001, Figure 2c). Ethanol’s effect was significantly enhanced in old rats, arising earlier (−19.4 ± 1.9 % at 0.04 × 10^−5^ M, *p* < 0.0001) and reaching −50.1 ± 2.4% at 0.9 × 10^−5^ M, *p* < 0.0001.

### 2.3. Relative Contributions of THC and Ethanol in Mitochondrial Respiration Impairments

Based on these data, we aimed to differentiate THC- and EtOH-specific effects on mitochondrial respiration.

**Figure 3:**
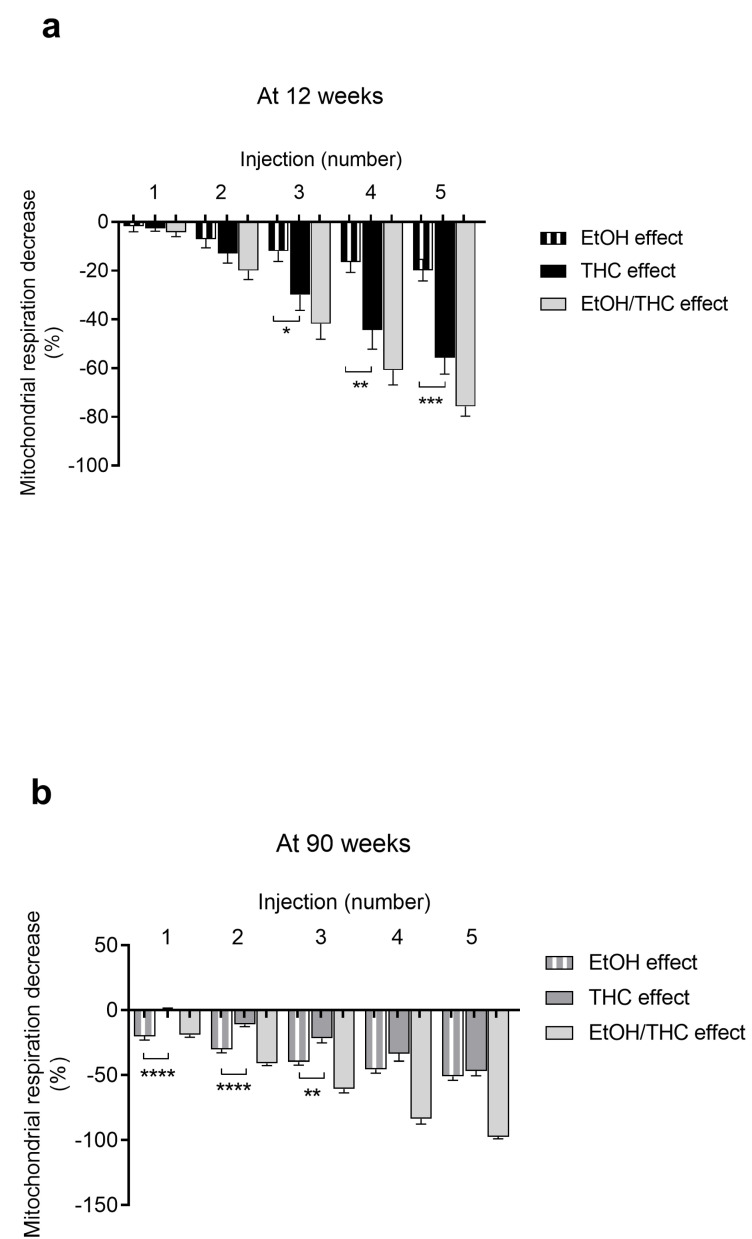
THC and EtOH contributions to mitochondrial respiration decrease in young (**a**) and old (**b**) rats. Values are means ± SEM. n = 6 for 12-week group with 12 runs, n = 3 for 90-week group with 8 runs. * *p* < 0.05, ** *p* < 0.01, *** *p* < 0.001, **** *p* < 0.0001 vs. EtOH effect. EtOH: ethanol. THC: tetrahydrocannabinoid.

In young cardiac mitochondria, the decrease in mitochondrial respiration due to THC was greater than that due to EtOH alone. The difference was significant (−55.8 ± 6.7 vs. −19.8 ± 4.4%, *p* < 0.001) at the fifth injection, and thus the impaired mitochondrial respiration appeared mainly related to THC (Figure 3a).

In old rats, the decrease in mitochondrial respiration was mainly due to ethanol at lower doses. At higher doses, both THC and ethanol contributed similarly. Thus, the maximal decrease in oxygen consumption of −97.6 ± 1.4% (THC + ethanol) was due to 46.7 ± 3.7% of THC and 50.9 ± 3.0% of ethanol alone, corresponding to the fifth injection. The enhanced mitochondrial dysfunction observed in old versus young rats can therefore be ascribed to THC and ethanol additive effects (Figure 3b).

### 2.4. THC, Unlike EtOH, Mainly Increased H_2_O_2_ Production in Old Rats

**Figure 4:**
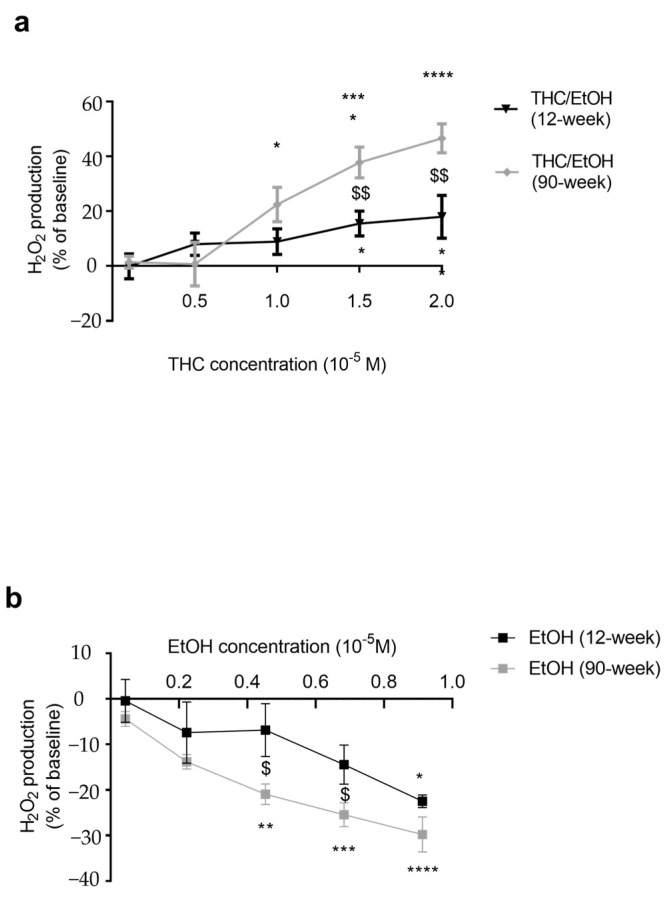
Dose–response effect of THC (**a**) or EtOH (**b**) on the cardiac mitochondrial hydrogen peroxide (H_2_O_2_) production, depending on the age. (**a**): Cardiac mitochondrial H_2_O_2_ production. Mitochondria were exposed to increasing THC doses. (**b**): Mitochondria were exposed to increasing EtOH doses corresponding to the increasing THC doses. Values are means ± sem. n = 2 for 12-week group with 4 runs and n = 3 for 90-week group with 8 runs. * *p* < 0.05, ** *p* < 0.01 *** *p* < 0.001, **** *p* < 0.0001 vs. baseline. Comparisons between groups: $ *p* < 0.05, $$ *p* < 0.01. EtOH: ethanol. THC: tetrahydrocannabinoid.

Mitochondrial H_2_O_2_ production was largely increased with THC exposure, particularly in old rats (+17.9 ± 7.8 vs. +46.6 ± 5.3% at 2 × 10^−5^ M, *p* < 0.01) (Figure 4a).

We also investigated an eventual role of ethanol per se on mitochondrial production of reactive oxygen species. EtOH decreased H_2_O_2_ production by -29.8 ± 3.8% (*p* < 0.0001) in old rats, and by −22.5 ± 1.4% (*p* < 0.05) in young rats at 0.9 × 10^−5^ M, corresponding to the 2 × 10^−5^ M dose of THC (Figure 4b). 

## 3. Discussion

This first report comparing the potential effects of THC on cardiac mitochondrial function in young and old rat hearts (corresponding to approximatively 16 and 70 years in humans) demonstrates that THC reduces cardiac mitochondrial respiration dose-dependently. Such THC-related impairment was associated with increased oxidative stress, as inferred from the concomitant measurement of H_2_O_2_ production. Interestingly, the increased susceptibility of cardiac mitochondria to THC in aged hearts also appeared to be potentially related to ethanol, since ethanol per se impaired mitochondrial respiration, mainly in older rats.

Chiu et al. analyzed the effect of THC on oxygen consumption in several tissue homogenates, including the heart. They observed reduced oxygen consumption [24]. Accordingly, previous data showed that THC can decrease cardiomyocytes’ mitochondrial respiration, likely acting through the mitochondrial respiratory complex I [31,32]. Further, unlike Salimi et al. in the setting of aluminum phosphide-induced cytotoxicity and Lu et al. showing that activation of cannabinoid receptors protects the mitochondrial function of cardiac myocytes exposed to pro-hypertrophic agonists [33,34], Athanasiou et al. observed that THC impairs mitochondrial respiration and increases oxidative stress [35]. Further, demonstrating that THC might be useful to inhibit oral cancer cells’ respiration, Whyte et al. also observed that THC impaired the mitochondrial respiration of beef hearts [36]. Consistent with these findings, in our study, the THC-related decrease in mitochondrial respiration was associated with an increased oxidative stress, as inferred from the concomitant augmentation of H_2_O_2_ production. Such an increase also suggests that mitochondria are a main source of ROS during cannabis exposure, as already observed in normal skeletal muscle [37]. Taken together, although likely depending on *Cannabis sativa* strains, these data strongly support that THC can reduce cardiac mitochondrial respiration, supporting that such a mechanism might participate in the worse prognosis of myocardial infarction in patients taking THC, together with previously described increased myocardial oxygen demand via increased heart rate and a simultaneous decrease in coronary blood flow [13,38,39].

Aging is known to decrease mitochondrial function, including mitochondrial respiration likely associated with increased oxidative stress. However, this is controversial, and like in this study, previous reports have demonstrated that mitochondrial respiration values might be unchanged by aging alone [40,41,42,43]. Nevertheless, in view of the increased susceptibility of old cardiomyocytes to ischemic or toxic [44,45] insults, we investigated whether age would enhance THC’s deleterious effects. This was the case and might be due to an additive action of ethanol on THC’s effect. Indeed, ethanol per se, used here as a solvent, specifically impaired mitochondrial respiration in older rats, likely participating in the enhanced mitochondrial dysfunction observed in older hearts. Accordingly, although data are rare, previous reports demonstrated that acute alcohol exposure impairs cardiac mitochondrial functions [46,47], and that chronic plus binge ethanol use induces myocardial mitochondrial dysfunction and oxidative stress [48]. Similarly, a synergistic deleterious effect was observed when associating alcohol with amphetamines [49]. The mechanisms involved still need to be investigated, but although increased H_2_O_2_ production has been reported with ethanol, results are controversial, and like in our study, ethanol was also recently reported to reduce H_2_O_2_ bioavailability in aortic cells [50,51].

## 4. Methods

### 4.1. Study Design

Experiments were performed on 12-week- (n = 6; 12 runs) and 90-week-old (n = 3; 8 runs) male Wistar rats (Janvier, Le Genest-St-Isle, France). Animals were housed at 22 ± 2 °C, with a 12 h light–dark cycle, water and food ad libitum, and with an enriched environment. This investigation was carried out in accordance with “the Principles of laboratory animal care” and respected the Guidelines of the European Union (86/609/EU) and the Committee for the Care and Use of Laboratory Animals (Cremeas, France, décret 2013-118, article. R. 214-89).

Rats were anesthetized with 3% isoflurane in an induction chamber (Minerve, Esternay, France) and then decapitated. The heart was excised and the left ventricle was immediately placed in an ice-cold isolation buffer. To investigate the dose–response effect of THC on mitochondrial respiration, synthetic THC (diluted 25 mg/mL in ethanol, Sigma Aldrich, St. Louis, MO, USA) was injected in the respiration chamber at the following concentrations: 0.1 × 10^−5^, 0.5 × 10^−5^, 1 × 10^−5^, 1.5 × 10^−5^, and 2 × 10^−5^ M, as inferred from our previous data [23]. Organic solvents are known to impair mitochondrial respiration, and Syed et al. studied the toxicity of ethanol, methanol, and acetonitrile on oxidative phosphorylation, showing that methanol has an IC50 at 8.3% (*v*/*v*), ethanol at 4.6%, and acetonitrile at 2.1% [52]. We therefore chose ethanol since, unlike the other solvents, ethanol is consumed by humans (and very often together with THC), allowing us to investigate such a combination. Further, methanol is particularly dangerous; a single sip of concentrated methanol can cause serious poisoning.

To determine the specific effect of ethanol, the solvent was used on its own at concentrations ranging from 0.04 × 10^−5^ M to 0.9 × 10^−5^ M, corresponding to the doses injected with THC (i.e., from the first to the fifth injection: 0.04 × 10^−5^ M, 0.2 × 10^−5^ M, 0.4 × 10^−5^ M, 0.7 × 10^−5^ M, and 0.9 × 10^−5^ M for EtOH alone).

### 4.2. Parameters Determined 

#### 4.2.1. Mitochondrial Extraction and Respiration 

Extraction was performed by sequential centrifugation. Briefly, the heart was washed and homogenized with a gentle MACS Dissociator (Miltenyi Biotec, Bergisch Gladbach, Germany). The homogenate was centrifuged at 3000 rpm for 3 min at 4 °C. Then, the supernatant was centrifuged at 8000 rpm (4 °C, 10 min). Mitochondria were washed and centrifuged at 11,000 rpm (4 °C, 5 min). Finally, mitochondria were suspended with an ice-cold buffer (50 mM Tris, 70 mM sucrose, and 210 mM mannitol, pH 7.4 at +4 °C), and protein content was quantified with a Bradford assay.

Cardiac mitochondrial respiration was determined using a high-resolution oxygraph (Oxygraph-2K, Oroboros instruments, Innsbruck, Austria) containing 2 Clark-type electrodes. An amount of 0.1 mg mitochondria was placed in 2 mL of respiration buffer (Miro5+creatine: EGTA 0.5 mM, MgCl2 3 mM, K lactobionate 60 mM, taurine 20 mM, KH2PO4 10 mM, HEPES 20 mM, sucrose 110 mM, BSA 1 mg/mL, creatine 20 mM). Mitochondrial respiration was explored in OXPHOS CI by injection of glutamate (10 mM), malate (2.5 mM), and ADP (2 mM) at 37 °C with continuous stirring. 

#### 4.2.2. Mitochondrial H_2_O_2_ Production

We determined mitochondrial H_2_O_2_ production using Amplex Red with an O2K-Fluo LED2-Module. Briefly, when Amplex Red (20 µM) found a molecule of H_2_O_2_, the horseradish peroxidase (1 U/mL) metabolized this couple on a fluorescent molecule, resorufin (563 nm/587 nm) [53]. The mitochondrial substrates added were glutamate, malate, and ADP, in the same concentrations as described in the methods for mitochondrial respiration analysis. Data were expressed in percentage of the control, which was the ADP injection without THC or ethanol.

## 5. Statistical Analysis

All data were expressed as mean ± standard error of the mean (SEM). The statistical analyses were performed using Prism software (GraphPadPrism 8.4.3, GraphPad Software, San Diego, CA, USA). After checking normality with the Shapiro–Wilk test, one-way ANOVA was performed with the Dunnett post hoc test to analyze the parameters’ evolution following THC or vehicle exposures. For the samples, following a normality test, a Student’s two-tailed t-test was used for group comparisons, and for other comparisons, a Mann–Whitney test was performed. A *p*-value < 0.05 was considered statistically significant.

## 6. Conclusions

In summary, THC, the main component of cannabis, directly impairs cardiac mitochondrial respiration dose-dependently. The simultaneous increase in ROS supports a participation of oxidative stress, and thus mitochondria-targeted antioxidants might be a useful therapeutic approach in this setting. Interestingly, THC’s effect was enhanced in older cardiac mitochondria when using alcohol concomitantly.

Therefore, although further studies are needed before translating these experimental data to human beings, further caution should be warranted when using cannabis and alcohol together. Hence, besides the need to favor the prevention of the simultaneous use of cannabis and alcohol whatever the age, these data highlight the critical importance of evaluating the individual benefits and risks of medical cannabis in older patients [54,55].

## Data Availability

Data contained within the article.
